# Conservation and Captive Breeding of the Asian Houbara Bustard (*Chlamydotis macqueenii*)

**DOI:** 10.3390/biology15110884

**Published:** 2026-06-03

**Authors:** Hanan Al-Khalaifah, Afaf Al-Nasser

**Affiliations:** Environment and Life Sciences Research Center, Kuwait Institute for Scientific Research, P.O. Box 24885, Kuwait City 13109, Kuwait

**Keywords:** Asian houbara bustard, captive breeding, conservation, reproductive technologies, restocking, genetic diversity

## Abstract

The Houbara Bustard (*Chlamydotis macqueenii*) is a vulnerable species found in arid and semi-desert regions of the Middle East and Central Asia. In recent decades, Houbara populations have significantly declined due to overhunting, habitat loss, and commercial development. As a result, the International Union for Conservation of Nature (IUCN) has classified this species as Vulnerable. This review examines global conservation efforts and breeding management strategies for the Asian Houbara Bustard, with particular focus on captive breeding programs established in Saudi Arabia and the United Arab Emirates. The study evaluates key reproductive technologies, including artificial insemination and other assisted breeding techniques, and examines the genetic and behavioral risks associated with captive breeding. Findings from the reviewed studies indicate that, while captive breeding programs have achieved notable production goals, they also present critical challenges for conservation. Effective long-term conservation therefore requires integrating reproductive technologies with habitat protection, careful management of genetic diversity, and the establishment of monitoring systems to ensure sustainable population recovery.

## Simple Summary

The Houbara Bustard *(Chlamydotis macqueenii*) is a vulnerable desert species that has experienced major population declines due to hunting pressure and habitat degradation across the Middle East and Central Asia. Over the past 30 years, conservation programs—particularly in Saudi Arabia and the United Arab Emirates—have focused on captive breeding and release initiatives that have produced thousands of birds. Although these programs have achieved significant progress in reproductive technologies, including improved artificial insemination and hatchability rates, challenges remain. Captive-bred birds often show reduced survival and altered behaviors such as weakened migration and predator avoidance, as well as potential genetic changes from long-term captivity. These findings indicate that captive breeding alone cannot ensure species recovery. Effective conservation requires integrating breeding programs with habitat protection, genetic management, and post-release monitoring to support sustainable Houbara populations.

## 1. Introduction

Avian species represent the most diverse class of terrestrial vertebrates, with over 11,000 recognized species worldwide playing critical ecological roles as pollinators, seed dispersers, and apex predators [1]. The conservation and genetic improvement of indigenous chicken breeds and non-galliform avian species—including quail, waterfowl, bustards, and ratites—represent critical priorities for enhancing global biodiversity and preserving the genetic diversity of domestic and wild bird populations [2,3]. Among these, the Houbara bustard (*Chlamydotis* spp.) merits particular attention due to its conservation status and cultural significance. The Houbara bustard is a medium-sized bird belonging to the family Otididae and comprises two recognized species: The African houbara bustard (*Chlamydotis undulata*) and the Asian houbara bustard (*Chlamydotis macqueenii*). Otis macqueenii was proposed by John Edward Gray in 1834 for a bustard from India drawn by Thomas Hardwicke. It was long regarded as a subspecies of the African houbara Chlamydotis undulata*, but was classified as a distinct species in 2003 [4]. The African Houbara bustard (*Chlamydotis undulata*) has two subspecies: one found in North Africa and the other in the Canary Islands. Most African Houbara bustards are resident populations occurring in Mauritania, parts of North Africa, and the Canary Islands. In contrast, the Asian species, commonly known as Macqueen’s bustard, shows a greater tendency for seasonal migration compared with the African houbara bustard [4].

Historically, *C. macqueenii* was treated as a subspecies of *C. undulata*, but it was later recognized as a separate species based on morphological and genetic differences [5]. Both species were previously assessed together under the name *Chlamydotis undulata* and were classified as Vulnerable (VU) on the IUCN Red List due to long-term population declines. Following the taxonomic split, both species were independently assessed and retained the Vulnerable (VU) status because of their continuing population decline and ongoing threats [6,7].

The Houbara bustard holds significant traditional value in Gulf countries because of its importance as a desert bird and its historical role in falconry [8]. In the past, houbara bustards bred widely across the Arabian Peninsula, with early records of breeding in Kuwait, Saudi Arabia, Iraq, and Oman. However, populations have experienced a long-term historical decline, largely driven by overhunting and extensive habitat degradation. Additional pressures include commercial development, agricultural expansion, aquaculture activities, energy production, mining, and transportation infrastructure development [9,10,11].

To address these threats and support population recovery, several captive breeding and conservation programs have been established in the Middle East, particularly in Saudi Arabia and the United Arab Emirates (UAE) [12]. The UAE initiated one of the earliest captive breeding programs in 1977 at Al Ain Zoo, with the objective of restocking regional houbara populations that had significantly declined. Later, the International Fund for Houbara Conservation (IFHC) was established in 2006 in the UAE to support a global conservation initiative through breeding, research, and release programs aimed at increasing houbara populations in the wild. As part of these efforts, several breeding and research centers have been established in the UAE, successfully increasing houbara production through advanced captive breeding programs [13].

Despite these efforts, houbara reproduction presents several biological challenges. Breeding is strongly seasonal, and even within the breeding season the reproductive performance of both males and females is limited. Furthermore, knowledge of the reproductive physiology of both sexes remains relatively limited [14,15]. Therefore, improving reproductive performance is critical for enhancing captive breeding success. Several reproductive technologies, including artificial insemination, in vitro fertilization, and surrogate eggshell techniques, have been explored to improve production efficiency. For example, Wernery et al. [16] attempted to apply primordial germ-cell technology using chicken embryos to increase houbara production; however, the results were limited.

In Kuwait, conservation of desert wildlife receives considerable attention. Improving houbara production and strengthening conservation efforts in Kuwait and the surrounding region are key priorities for Al-Diwan Al-Amiri and the Kuwait Institute for Scientific Research (KISR). As part of these initiatives, KISR has developed a comprehensive plan to establish a Houbara Breeding Center (HBC) in Kuwait. The aim of this center is to improve the production of the Asian houbara bustard through captive breeding, restocking, monitoring, and research programs. In parallel, KISR has prepared a research program focusing on houbara reproductive physiology and assisted reproductive technologies (ART), such as artificial insemination, sperm cryopreservation, and in vitro fertilization, to enhance breeding success.

Given the continuing decline of wild populations and the increasing reliance on captive breeding programs, it is essential to critically review current conservation strategies, reproductive technologies, and breeding management practices. This review therefore aims to evaluate global conservation efforts and breeding management strategies for the Asian houbara bustard, with particular emphasis on captive breeding programs in the Middle East and their role in supporting long-term species conservation.

## 2. Materials and Methods

Electronic databases (Web of Science, Scopus, PubMed, Science Direct, and Google Scholar) and institutional repositories (Kuwait Institute for Scientific Research (KISR), International Fund for Houbara Conservation (IFHC), and the National Wildlife Research Center (NWRC) were searched for peer-reviewed articles, conference proceedings, and technical reports published in English. The literature search focused on studies related to houbara bustard conservation, captive breeding programs, reproductive biology, and reproductive technologies such as artificial insemination and assisted breeding techniques.

## 3. Species Description and Taxonomy

### 3.1. Bustards (Family Otididae): General Background

Family Otididae currently includes 26 species, as in the past; however, this number reflects taxonomic revisions over time. According to the IOC World Bird List v15.1 [17] bustards are medium- to large-sized terrestrial birds that inhabit open plains and semi-desert regions. They are distributed mainly in Europe, Asia, Africa, and Australia and belong to the order Otidiformes and the family Otididae, which comprises 11 genera and 26 species [18,19].

### 3.2. Taxonomy

The Houbara Bustard (*Chlamydotis undulata*) is a medium-sized, polytypic Palearctic species that inhabits steppe and semi-desert areas with open [9,20] and shrub-covered plains, ranging from the Canary Islands across North Africa to the Middle East and Central Asia [21,22]. Three subspecies of the Houbara Bustard have historically been recognized: the nominate North African race, *Chlamydotis undulata undulata*, which occurs across North Africa from Mauritania to Egypt; *Chlamydotis undulata fuerteventurae*, the Canary Islands race and the rarest subspecies, found mainly on the islands of Fuerteventura and Lanzarote; and the Asian race, *Chlamydotis undulata macqueenii*, which ranges from Egypt east of the Nile through the Sinai Peninsula and the Arabian Peninsula to Pakistan, Afghanistan, Kazakhstan, and Mongolia [20,21,22].

**Figure 1:**
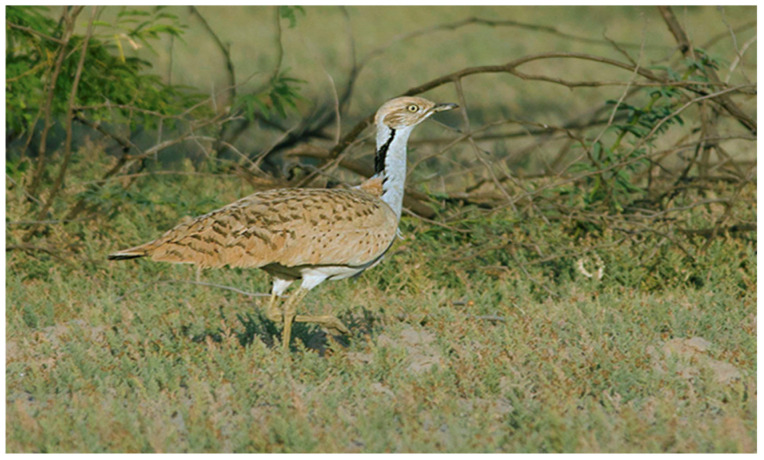
Asian Houbara Bustard (*Chlamydotis macqueenii*) [28].

**Figure 2:**
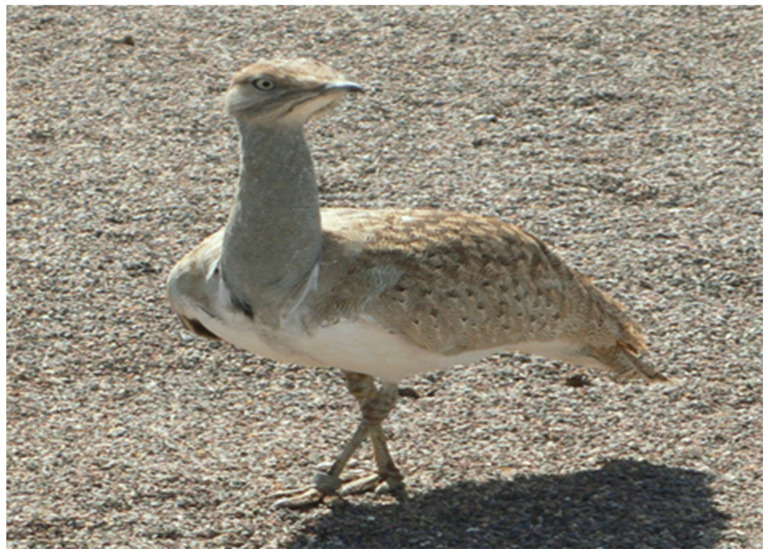
African Houbara Bustard (*Chlamydotis undulata*) [29].

Recent studies have proposed a taxonomic revision of the genus *Chlamydotis* into two distinct species. The Asian Houbara (*Chlamydotis macqueenii*) and the African Houbara (*Chlamydotis undulata*), including its subspecies *Chlamydotis undulata fuerteventurae*, are closely related species distributed across Asia and North Africa, respectively [21,23,24,25,26,27]. These species differ primarily in body size, plumage characteristics, and courtship display behavior. Figure 1 presents the Asian Houbara, while Figure 2 shows the African Houbara.

The Asian Houbara (*Chlamydotis macqueenii*) is larger and much paler than the African Houbara (*Chlamydotis undulata*) [4]. Table 1 compares the two species, focusing on specific features such as morphology, plumage patterns, and other key characteristics.

## 4. Distribution and Occurrence

**Figure 3:**
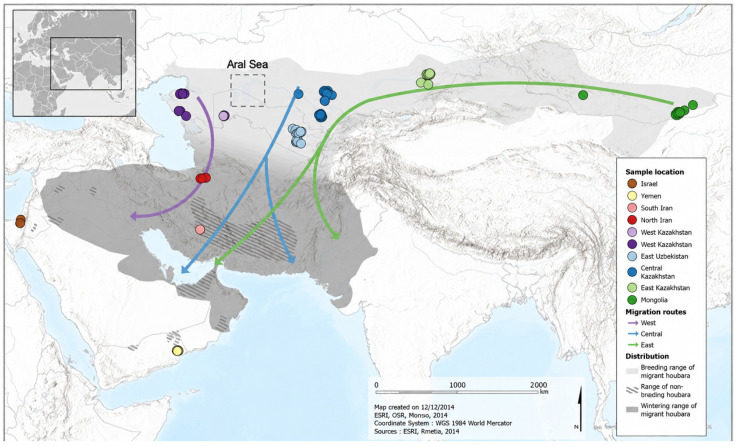
Distribution range of migratory populations (solid grey shading) and resident populations (dark grey cross-hatched shading) of the Asian Houbara (*Chlamydotis macqueenii*), showing sampling sites and the three primary migration pathways (indicated by colored arrows) [34].

The distribution range of the Asian houbara (*Chlamydotis macqueenii*) extends from the Arabian Peninsula and Central Asia eastward to Mongolia and northwestern China. Its range includes Israel, Palestine, Jordan, Syria, Saudi Arabia, Yemen, Oman, United Arab Emirates, Bahrain, Qatar, Iraq, Kuwait, Iran, Afghanistan, Pakistan, India, Turkmenistan, Uzbekistan, Tajikistan, Kyrgyzstan, Kazakhstan, and Mongolia. Limited or sporadic occurrences have also been reported from Azerbaijan, Armenia, southern Russia, and Turkey [31]. The distribution boundary between the Asian houbara (*C. macqueenii*) and the North African houbara (*Chlamydotis undulata*) is generally considered to occur around the Sinai Peninsula. While occasional records of *C. macqueenii* may occur in the Sinai and Negev Desert regions, the North African species, *C. undulata*, predominates west of Sinai and across North Africa [32,33]. Figure 3 shows the global distribution map of the Houbara Bustard.

Field studies conducted by the Aridland Agriculture Department (AAD) of the Kuwait Institute for Scientific Research (KISR) wildlife team have indicated that the Asian Houbara bustard (*Chlamydotis macqueenii*) is a winter visitor to Kuwait [35,36,37,38]. Historical records from the early twentieth century suggested the possibility of breeding within the region. Reports by Ticehurst, Buxton, and Cheesman [39] indicated that Houbara eggs collected in Kuwait were deposited in the British Museum. Additional historical observations from Dickson and other early naturalists also mentioned occasional nesting records in northwestern areas of Kuwait and nearby desert regions. However, contemporary observations suggest that regular breeding in Kuwait is unlikely, possibly due to increased anthropogenic pressures, particularly hunting and habitat disturbance. Field observations previously suggested that the Asian Houbara Bustard (*Chlamydotis macqueenii*) bred in Kuwait; however, current evidence indicates that regular breeding is unlikely. This change is thought to be associated with increased anthropogenic pressures, particularly hunting activities involving four-wheel-drive vehicles [36].

## 5. Ecology of the Houbara Bustard

### 5.1. Habitat

The Houbara Bustard is adapted to desert environments, preferentially inhabiting undulating arid plains, steppes, and semi-desert habitats, often with little cover except for open or scattered desert shrubs [40,41,42,43,44,45]. Vegetative cover consists of moderate or sparse perennials, primarily grasses, herbs, and shrubs, but sometimes includes larger bushes and trees. Typical plant associations include Artemisia, Haloxylon, and Salsola stands, cactoid Euphorbias, and Eremopterix grasses. *Asphodelus microcarpus*, *Noaea mucronata*, *Zilla spinosa*, and *Anabasis salsa* may also be present. Within their distribution zone, annual rainfall rarely exceeds 200 mm. When undisturbed, houbara may also use cultivated areas [46] such as alfalfa fields (*Medicago sativa*), as observed regularly on the Canary Islands and in birds kept in the Mahazat as-Sayd reserve in Saudi Arabia.

### 5.2. Diet

The Asian Houbara Bustard (*Chlamydotis macqueenii*) is an opportunistic omnivore whose diet reflects the seasonal availability of plant and animal resources [41,43,44]. Plant material constitutes an important component of the diet, particularly during winter and early spring, and includes fruits, seeds, shoots, leaves, flowers, and bulbs from genera such as *Ziziphus*, *Salsola*, *Lycium*, *Launaea*, and *Allium*. Cultivated crops, including beans, peas, alfalfa, and mustard, may also be consumed when available [47]. During spring and summer, the diet includes a greater proportion of animal prey, particularly invertebrates such as grasshoppers, weevils, termites, locusts, beetles (*Tenebrionidae*, *Scarabidae*, *Cantharidae*), caterpillars, scorpions, spiders, and ants, as well as snails and small vertebrates including snakes, lizards, and geckos [43,44]. Juveniles rely predominantly on insects and other small invertebrates. Reports regarding drinking behavior are inconsistent; some observations indicate frequent drinking, whereas others suggest that the species can obtain sufficient moisture from its diet [47].

### 5.3. Behavior and Breeding Biology

The Houbara has been described as monogamous [47], polygamous and promiscuous. Mendelssohn et al. [45] reported that pair bonds form in the spring and that males are seen in the vicinity of females and chicks. Houbara males have never been seen incubating eggs or feeding chicks, suggesting that they are indeed truly promiscuous.

Males attract their mates with an extravagant courtship display, which they perform at the same site each year. The display begins with a period of strutting and culminates with the male retracting his head within an ornamental shield of erected neck feathers and then running at speed in either a straight or curved line. The display is often accompanied by a series of subsonic booming calls [5]. In eastern Morocco, females breed from mid-February to mid-June and generally lay two to three eggs on the ground [48]. This information refers to the North African population studied in Morocco; nesting on the ground makes eggs and chicks particularly vulnerable to ground predators.

**Figure 4:**
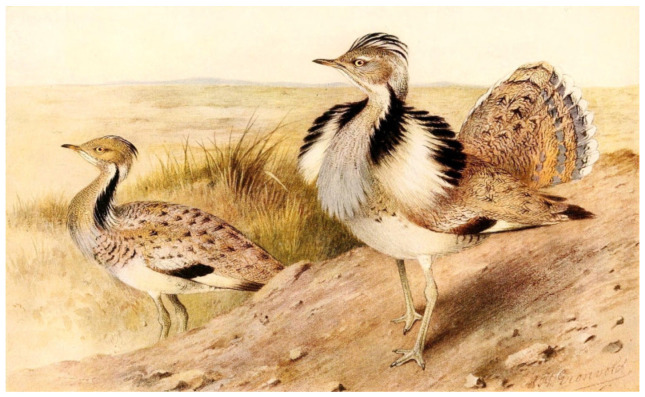
Illustration of a male in partial display with the ruff or collar erected [49].

At the beginning of the display sequence the male stands motionless; after some seconds, the elongated crown and neck-plumes are raised slowly up and forward, the wing-tips are lifted up slightly, and the tail is pressed down. The head is drawn back until it rests on the bird’s shoulders, and the breast and neck-feathers continue to rise, finally entirely covering the head. The bird, which was previously well camouflaged, suddenly becomes visible as a white spot over distances of at least two kilometers in open land, it now looks like a bird with a large white ball attached to its breast. After remaining in this posture for several seconds, the male begins to run in a straight line, in circles, or in a zigzag pattern, depending on the topography of the display ground. He runs with short steps, lifting the legs in an exaggerated manner. The male abruptly stops at the end of each run, jerking its head and neck forward once, uttering a low-pitched noise and then pulling its head and neck back again. The bird may then move back into its motionless starting posture, and relax and return to normal, or it might stand with erect feathers until the next display run. Most displays can be observed in the early morning and late afternoon. When a female appears at the display ground, the male begins a pre-copulatory display; it lowers the breast-feathers, approaches to directly in front of the female, and with the neck feathers still erected, swings its head and neck with forward-jerking movements, rapidly from right to left at about one-second intervals. When the female squats down, the male moves behind her, pecks her head and neck for several seconds and finally mounts her for copulation [5]. Figure 4 shows the illustration of a male Asian Houbara bustard (*Chlamydotis macqueenii*) performing a partial display, with the ruff (collar) feathers erected.

Although the nesting season of the Houbara is quite variable across the species’ range, most clutches are produced during the spring months. Egg-laying can occur between January and June with a peak in mid-March. Usually, males began to display by December and January. Houbara nests are shallow scrapes occasionally lined with vegetation that are situated on gentle slopes and elevated ground, rather than in depression. The slopes tend to be south facing, presumably to maximize isolation. Clutch size is usually two or three eggs, but occasionally one or five-egg-clutches are laid. Only one clutch is produced per season, but replacement clutches can be laid if the first clutch is destroyed. During exceptionally dry years houbara may not breed at all. Chicks are precocial and nidifugous at hatching, live food is offered to the chicks, bill-to-bill, accompanied by soft vocalizations from the female. At 2 or 3 days of age, females drop prey on the ground to be retrieved by the chicks. When 5 or 6 days old the chicks start to feed independently. After 11 days the wings and shoulder areas of the chicks are covered by contour feathers, and at 4 weeks the birds are fully feathered, although the wings and tail are still quite short. They start flying short distances at the age of 1 month but remain close to their mother for at least the next two months [50].

Limited information exists on the reproductive biology of this species and the potential for producing a significant number of chicks. Mating typically occurs most frequently during the spring months, although the exact timing can differ across the species’ range [50]. In Algeria, eggs can be found as early as November and as late as June [21]. The Houbara bustard is known to lay between one and four eggs on alternate days. As noted by Cramp and Simmons [21], wild Houbara bustards typically lay one clutch annually but may produce a replacement clutch if the initial one is unsuccessful. Two of the three subspecies, *C. u. undulata* and *C. u. macqueenii*, are bred in captivity at Taif. The first successful breeding occurred in 1989, resulting in 17 chicks. Subsequently, 55 and 49 chicks were produced in 1990 and 1991, respectively, and a total of 138 chicks were hatched in 1992. The low fertility rates from natural mating, which yielded a maximum of 30% viable eggs, led to the adoption of artificial insemination methods [14].

Artificial insemination represents a highly effective methodology in the controlled propagation of endangered avian species, particularly in instances where natural copulation proves inadequate in achieving satisfactory reproductive outcomes. The application of artificial insemination has demonstrated efficacy in domesticated avifauna such as turkeys, guinea fowl, chickens, mallards, and geese [14]; however, there exists a paucity of research on pertaining to wild bird species.

## 6. Conservation Status and Threats

According to the Convention on International Trade in Endangered Species of Wild Fauna and Flora (CITES) [51] and the IUCN Red List [30], many bustard species (family Otididae) are experiencing population declines due to habitat loss, hunting pressure, and other anthropogenic threats, highlighting the urgent need for conservation measures. The Asian houbara (*Chlamydotis macqueenii*), previously considered endangered in earlier assessments, is currently classified as Vulnerable (VU) on the IUCN Red List. Current IUCN assessments classify three species as Critically Endangered (CR)—*Ardeotis nigriceps*, *Houbaropsis bengalensis*, and *Sypheotides indicus*. Two species are listed as Endangered (EN)—*Neotis ludwigii* and *Otis tarda*. Three species are categorized as Vulnerable (VU)—*Chlamydotis macqueenii*, *Chlamydotis undulata*, and *Neotis nuba*. Four species are considered Near Threatened (NT)—*Eupodotis caerulescens*, *Eupodotis humilis*, *Neotis denhami*, and *Tetrax tetrax*. In contrast, *Ardeotis australis* is currently assessed as Least Concern (LC). These classifications emphasize the varying conservation status across the family Otididae and underline the importance of continued monitoring, habitat protection, and international cooperation to ensure the long-term survival of these species.

**Figure 5:**
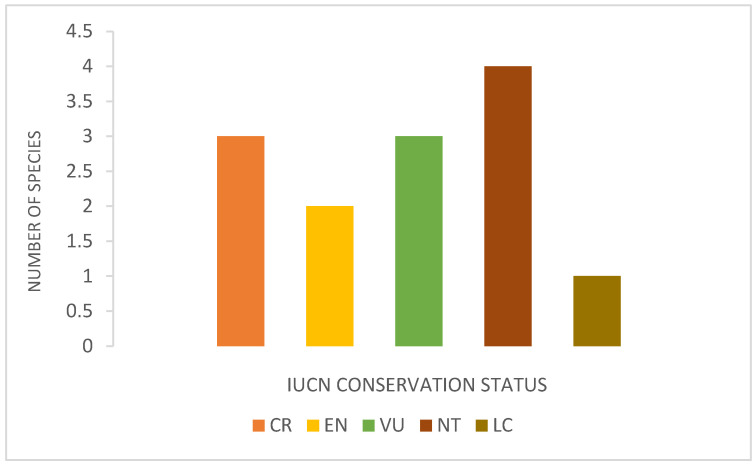
IUCN conservation status categories for Houbara bustard species. CR—Critically Endangered, EN—Endangered, VU—Vulnerable, NT—Near Threatened, LC—Least concern.

The official IUCN category for the houbara bustard is *Vulnerable* (*Vu*), which includes populations that are seriously depleted or decreasing due to overexploitation or other factors, and are at risk of becoming *Endangered* (*En*) if causal factors continue unchecked. During the last half of the 20th century, houbara bustard populations declined drastically throughout much of its range, and the species is now considered ‘*Endangered*’ in many countries [52]. The predominance of threatened categories within the family *Otididae* (Figure 5) underscores the urgent need for coordinated conservation action across range states.

Bustard species have demonstrated the capacity to adapt to anthropogenically modified environments, exhibiting population persistence and even demographic success in landscapes characterized by extensive management regimes. Such areas differ from intensive agricultural systems through the implementation of regulated hunting practices, minimal anthropogenic disturbance, reduced mechanization, limited agrochemical application, and restricted fencing infrastructure [53]. The Houbara’s role as the favored quarry of Arab falconers, along with severe habitat destruction due to overgrazing and human encroachment into once-remote desert areas, are the main reasons for the decline of houbara populations. During the last two decades, hunting of houbara has increased, due in part to the increased availability of fire-arms, but mainly due to increased pressure from Arab falconry parties [50]. With several dozen trained falcons, large numbers of Houbara can be killed by a single hunting party, exerting significant pressure on local populations [50]. The practice of falconry, especially the capture of birds in Pakistan and Iran for Arabian nations, continues to exert pressure on wild populations. Furthermore, the risk of collisions with power lines is increasing, particularly as renewable energy infrastructure grows in open areas preferred by this species [32]. Concerns over the threat of extinction of Houbara populations led to the establishment of captive breeding, propagation, conservation, and release programmes of houbara bustard in several countries and made the Houbara Bustard an ‘*umbrella* species’ in several conservation and protection programs of native wildlife fauna and flora.

Migratory Asian Houbara *Chlamydotis macqueenii* populations are facing a decline due to excessive licensed hunting, unregulated poaching, and trapping for trade [10,54,55]. Species survival hinges on sustainable management as a limited resource and necessitates coordinated efforts among traditional hunters, governments, and conservationists across its distribution. Nonetheless, the primary (effectively the sole) conservation action taken thus far has been the creation of captive breeding facilities in Kazakhstan, Uzbekistan, and Arabian nations to bolster populations and offset the loss of wild individuals due to hunting [33,56,57]. Furthermore, prior to a captive-bred Asian Houbara joining the breeding population, it must endure the post-release phase, later migration and overwintering, as well as additional time until reaching breeding age. Males of the Asian Houbara (*Chlamydotis macqueenii*) typically reach sexual maturity at 2–3 years of age, whereas females may become reproductively capable at approximately one year of age. However, successful nesting and chick rearing are generally observed after females reach around two years of age [58,59,60,61]. Additionally, although captive-bred Houbara released into the wild have been documented to nest under natural conditions, their reproductive success may be reduced due to the accumulation of maladaptive traits or the loss of advantageous learned behaviors, a pattern reported in other released species [62,63].

In addition, climate change is becoming a major factor affecting the species’ survival. Rising temperatures harm chick survival, and extreme weather conditions lead to more deaths. Nest predation and egg collection also limit reproductive success. Disturbances from vehicles and human activity in breeding areas can lower nesting success. These issues, along with the species’ slow life history and low reproductive rate, make it even more vulnerable to a decline in population [64].

The Asian houbara bustard (*Chlamydotis macqueenii*) is affected by multiple anthropogenic and environmental threats, with hunting and poaching identified as the dominant drivers of population decline. Hunting and poaching are estimated to contribute approximately 50–60% of total population pressure. Habitat degradation, driven by land-use change, infrastructure expansion, and overgrazing, accounts for approximately 20–25% of the impact. Trapping for trade contributes around 10–15%, particularly in regions such as Pakistan and Iran. Infrastructure-related mortality, including collisions with power lines, contributes approximately 5–10%, while other emerging threats such as climate change, nest predation, and human disturbance collectively represent <10% of the overall pressure [33,54].

**Figure 6:**
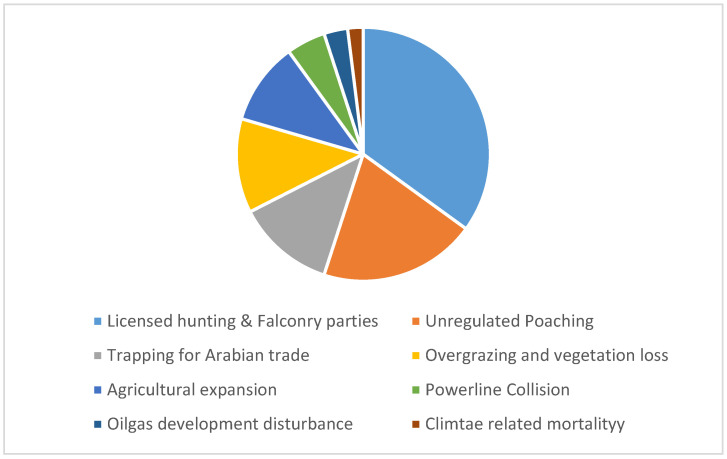
Relative contribution of major threats affecting *Chlamydotis macqueenii* across its range.

Although these proportions vary geographically and temporally, they provide a generalized representation of the relative importance of each threat category. These estimated contributions are illustrated in Figure 6 to provide a clearer visual understanding of their comparative impacts.

**Figure 7:**
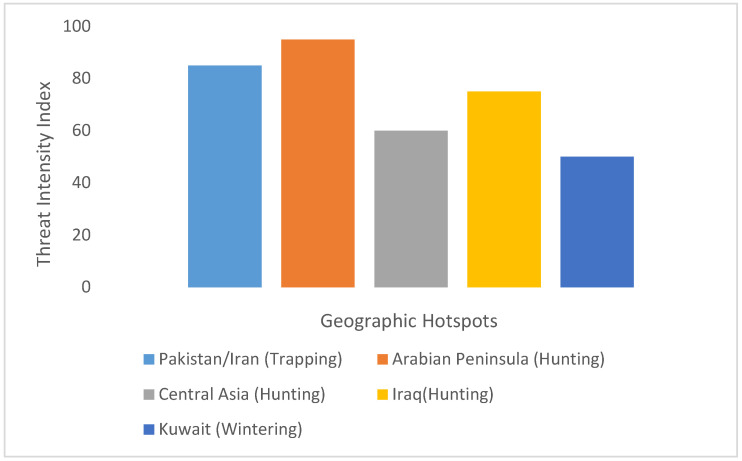
Regional Threat intensity of *Chlamydotis macqueenii* in Geographic Hotspots.

The regional variation in threat intensity indicates that the Arabian Peninsula experiences the highest pressure, primarily due to intensive hunting activities, followed by Pakistan and Iran, where trapping represents a major threat [33]. Figure 7 summarizes the regional threat intensity of the Asian Houbara Bustard (*Chlamydotis macqueenii*) across key geographic hotspots.

## 7. Conservation and Management of Houbara Birds

Captive breeding and conservation programs are key conservation strategies for restoring depleted Houbara Bustard populations in the Middle East and Central Asia. These programs integrate captive propagation, reproductive research, and carefully managed release schemes to enhance the recovery of the population. Captive breeding programs have been practiced in countries such as Saudi Arabia and the UAE, aiming to conserve Houbara birds. The Kingdom of Saudi Arabia has pioneered the conservation of endangered native wildlife species by establishing captive breeding programs through the National Commission for Wildlife Conservation and Development (NCWCD) in 1986. The NCWCD established the National Wildlife Research Center (NWRC) in Taif for the captive breeding of desert animals, including the Houbara Bustard. The aim was to establish a new Houbara population in the wild, in KSA, to replace the populations of resident Houbara eliminated by over-hunting and habitat loss [65]. Breeding success was first achieved in 1989, with the production of 17 chicks. Significant improvements in breeding techniques have resulted in a further increase in the number of chicks produced from both species of Houbara Bustards. The total number of Houbara chicks hatched since 1989 reached 1050 for the Asian species, with the combined total for both Asian and African species reaching 1550 chicks. The use of artificial insemination boosted fertility from 69.5% in 1992 to 90% in 1998. Additionally, hatchability increased from 53.7% to 75% over the same period [64]. After the females are artificially inseminated, eggs are collected and placed in machines that control temperature and humidity. Hundreds of eggs can be maintained in each incubator simultaneously. The hatchlings are sent to the raising building after three weeks of incubation, where any illnesses or weaknesses are treated. Dietary conditioning is gradually modified to promote natural foraging behaviors. After four weeks of rearing, the juveniles were placed in netting tunnels with shade, where they were given less water, mealworms, and alfalfa hay to help them get used to living bugs. They also began to interact less with humans. In this way, they become accustomed to the desert environment.

Other countries that established captive breeding of Houbara Bustards’ species in wildlife and biodiversity conservation are the National Avian Research Center (NARC) of United Arab Emirates, and the Emirates Centre for Wildlife Propagation (ECWP) in Morocco [12,66]. The research in KSA and UAE included restoration programs, habitat protection, ecological studies, biomedical research, public awareness programs, rehabilitation projects, contacts with local hunting organizations and falcon research groups for the sustainable use of houbara range, and international agreements to conserve the Houbara Bustard. Breeding houbaras in captivity is a difficult task given the paucity of knowledge about the ecology and biology of this species [67]. In addition, very little information is known about the reproductive system of both Houbara males and females [14,15].

The present research examined the literature regarding the Houbara Bustard’s conservation status over the past 30 years (1995–2024), as illustrated in Table 2.

The variation in conservation status reported by the same organizations (e.g., NWRC and IFHC in the UAE) reflects differences in assessment periods, geographic scope, and the use of earlier versus updated IUCN classification systems. Conservation status may change over time as new population data become available. Therefore, some reports classify the Houbara as Endangered based on earlier or regional assessments, whereas more recent global evaluations by the IUCN categorize the species as Vulnerable.

KISR’s approach differs from regional counterparts in three key respects. First, while NWRC and IFHC prioritize large-scale production for release and falconry, KISR integrates reproductive physiology research—specifically in vitro fertilization, surrogate shells, and primordial germ-cell techniques—as a core operational component rather than an ancillary activity. Second, KISR is positioned to implement genetic integrity and behavioral competence protocols from inception, avoiding the retrospective adaptations required by established programs after decades of captive breeding. Third, KISR’s initiative addresses Kuwait’s unique ecological context where houbara are currently winter visitors rather than breeding residents, necessitating careful reintroduction science rather than simple population supplementation.

Captive breeding has been widely used as a conservation management strategy for the Asian Houbara, aiming to support depleted populations while also supplying birds for hunting and falconry training. Initially bred in the 1970s [45], it required two decades before large-scale mass breeding became feasible in response to demand [68,72]. Through the use of artificial insemination techniques, the species is currently produced at an annual rate of tens of thousands, with substantial numbers being released into the wild [72]. This production methodology necessitates a high degree of docility among the breeding stock. Dolman et al. [72] further documented that captive-bred individuals in Asia and the Middle East exhibited significant levels of tameness, occasionally approaching vehicles and humans. The survival rate of captive-bred and subsequently released birds in the United Arab Emirates is notably low [70], and while the survival rate of juveniles released in Uzbekistan into late autumn approached that of their wild counterparts [73] their survival beyond their first full migration is significantly diminished (23% compared to 37%; [74]), with evidence indicating alterations in phenology [75]. Furthermore, apprehensions have been articulated concerning the release of captive-bred individuals exhibiting behaviors (such as resident versus migratory) or originating from geographic lineages that are misaligned with the respective release sites [74].

Saudi Arabia and the United Arab Emirates have undertaken extensive Houbara conservation and research initiatives across the Arabian region. These efforts include the establishment of captive breeding and restoration programs, habitat protection, ecological and biomedical research, public awareness campaigns, rehabilitation projects, collaboration with local hunting organizations and falconry research groups, and participation in international agreements aimed at the sustainable management and conservation of Houbara bustards. In this region, these initiatives primarily focus on the Asian Houbara (*Chlamydotis macqueenii*). In contrast, captive breeding and conservation programs in other countries target different Houbara species. For example, Morocco focuses on the African Houbara (*Chlamydotis undulata*), whereas Uzbekistan implements breeding and release programs primarily for the Asian Houbara (*Chlamydotis macqueenii*). Such captive breeding initiatives have been established as a strategy to support the long-term survival of Houbara populations [51].

## 8. Challenges Facing Current Breeding of *Chlamydotis macqueenii*

The main challenges in breeding *Chlamydotis macqueenii* are poor reproductive knowledge, low female breeding probability, weather-sensitive breeding, nest predation and abandonment, and the risk that captive breeding/release can cause domestication, genetic dilution, and weak post-release survival.

### 8.1. Reproductive Biology Constraints

Captive breeding of the migratory Asian houbara bustard (*Chlamydotis macqueenii*) is constrained by a complex set of interrelated biological, ecological, and management challenges. First, limited knowledge of the species’ reproductive biology—including endocrinology, courtship behaviour, sperm quality, and ovulation mechanisms—continues to restrict breeding efficiency and the optimization of assisted reproductive techniques [62]. Insufficient understanding of hormonal cycles and environmental cues such as photoperiod and social interactions limits the ability to reliably manipulate reproductive output under captive conditions [76,77,78,79,80,81].

### 8.2. Post-Release Mortality

Second, high post-release mortality remains a major limitation of reinforcement programs, with survival rates often insufficient to offset population declines driven by unsustainable hunting pressure [74]. Mortality during the post-release phase is frequently associated with inadequate adaptation to natural environments, migration challenges, and increased predation risk.

### 8.3. Genetic and Phenotypic Alterations

Third, captive breeding influences numerous heritable traits through founder effects, genetic drift, relaxation of natural selection, and inadvertent adaptation to captive environments. These processes result in morphological, physiological, endocrine, metabolic, thermoregulatory, behavioral, and temperamental changes, many of which are maladaptive under natural conditions [69,82,83,84,85,86]. Such adaptations are predominantly maladaptive 493 when animals are reintroduced into wild settings [83]. Quantitative genetic models indicate that even under weak selection differentials, reinforcement programs can alter wild phenotypes when captive-bred individuals are introduced into natural populations [87].

Furthermore, captivity often selects for elevated fecundity levels that diverge from wild reproductive optima [82,88,89]. Immunocompetence may decline due to inbreeding, founder effects, and genetic drift, while captive environments may favor immune traits that are maladaptive in natural pathogen landscapes, thereby reducing resistance upon release [90,91,92,93]. Behavioral deficiencies, including impaired foraging, locomotion, territoriality, and predator avoidance, are also frequently observed, resulting in reduced survival and reproductive success relative to wild conspecifics [93,94,95,96,97].

### 8.4. Behavioural and Ontogenetic Impairments

Captive conditions can promote genetic domestication, habituation to humans, and impaired ontogenetic development, often leading to the loss of natural behaviors and altered temperament, including increased docility [73]. Additionally, the absence of parental learning during captive rearing can further reduce reproductive success in released individuals [97,98]. Although predator-aversion training has demonstrated some success, its effectiveness remains inconsistent and may involve substantial logistical and ethical constraints [95,99,100].

### 8.5. Epigenetic and Rapid Genetic Adaptation

Captive breeding may also induce epigenetic and ontogenetic modifications arising from environmental and physiological influences during early development, leading to heritable phenotypic and behavioral changes across generations [73]. Rapid genetic adaptation to captivity has been documented despite mitigation efforts, with measurable changes in gene expression and reproductive traits occurring within only a few generations [81,86,89,101,102].

### 8.6. Ecological and Management Concerns

**Figure 8:**
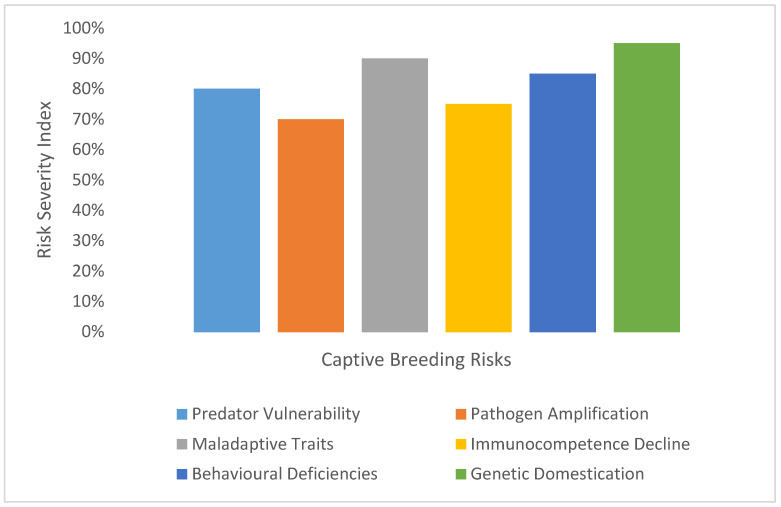
The multifaceted risks associated with captive breeding programmes.

Finally, broader ecological and management concerns persist. Supplementation programs may inadvertently increase hunting pressure and illegal harvest, as well as facilitate predator learning and search image formation [103]. High-density captive conditions also increase the risk of pathogen amplification and transmission to wild populations, as observed in bustard conservation programs [104]. Collectively, these factors contribute to ongoing scientific debate regarding the long-term conservation efficacy of captive breeding for this species [76]. Figure 8 summarizes major challenges associated with captive breeding of *Chlamydotis macqueenii*. Reproductive limitations and high post-release mortality represent the most critical constraints, followed by genetic and behavioral challenges that affect long-term population viability.

## 9. Measures to Address Captive Breeding Challenges

To address the multifaceted challenges associated with captive breeding of *Chlamydotis macqueenii*, a combination of integrated research, genetic, ecological, and management strategies is required. Enhancing reproductive success necessitates comprehensive investigation of endocrine regulation, reproductive physiology, and breeding behavior in both sexes, alongside the refinement of assisted reproductive technologies such as artificial insemination and in vitro fertilization.

Genetic management remains central to maintaining population viability. Reinforcement programs should explicitly consider the biogeographical and genetic structure of both source and recipient populations to prevent genetic homogenization, introgressive hybridization, and the erosion of locally adapted gene pools [80,105]. The delineation and management of evolutionarily significant units (ESUs), despite ongoing methodological debate, are essential for preserving adaptive diversity [81]. This requires rigorous founder selection, controlled breeding strategies, and periodic genetic monitoring to minimize inbreeding and unintended introgression [106,107,108,109]. In this context, comprehensive demographic and genetic profiling—including whole-genome sequencing and genome-scale comparisons of both wild and captive populations—is critical to ensure alignment between them and to quantify domestication effects, including changes in immunocompetence, temperament, and behavioral traits [73].

Such profiling should also be used to evaluate the relative contributions of genetic inheritance and learning to key survival traits such as predator avoidance, as well as to assess the extent of introgression into wild populations. Furthermore, systematic measurement and modelling of ecological, behavioral, and genetic impacts of released individuals are required, including comparative assessments of fitness, survival, and reproductive success between captive-bred and wild conspecifics. Although such data are essential for evidence-based management, their limited public availability remains a constraint on evaluating program effectiveness [73].

To mitigate domestication effects, it is important to limit the number of captive generations and cautiously incorporate wild individuals into breeding populations. However, while periodic infusion of wild stock may slow genetic divergence, it does not fully eliminate domestication-related changes, particularly where introgression into wild populations has already occurred [83,87,110].

Improving post-release survival requires the implementation of pre-release conditioning protocols, including habitat acclimatization and behavioral training. Predator-aversion training may be applied selectively, although its effectiveness remains variable and resource-intensive [94,99,100]. In parallel, strict biosecurity measures are essential to minimize disease transmission risks, including health screening, pathogen surveillance, and the regulation of captive population densities.

At a broader scale, conservation strategies should adopt a landscape-level and ecologically informed approach, ensuring that release programs align with regional environmental conditions and species-specific ecological requirements [76]. This includes the establishment of non-hunting zones and the implementation of scientifically determined harvest quotas for both released and wild individuals, which are critical for maintaining sustainable population dynamics. Public awareness and outreach initiatives targeting hunting communities are also necessary to reduce unsustainable exploitation and promote long-term conservation objectives.

**Figure 9:**
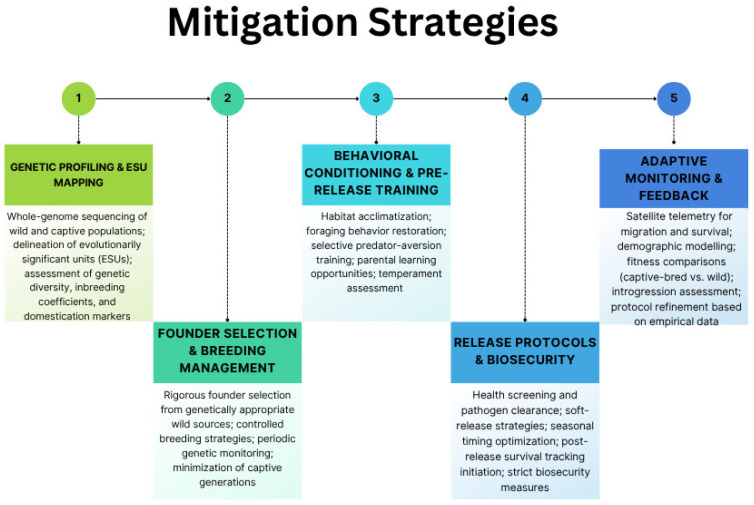
Five-stage mitigation framework for *C. macqueenii* conservation.

Finally, given the substantial investment in existing breeding programs, a strategic reorientation toward more holistic conservation planning is recommended. Depending on empirical evidence, captive breeding may need to assume a more limited or geographically targeted role, such as supporting “put-and-take” systems within managed hunting concessions, while prioritizing the recovery of self-sustaining wild populations in protected landscapes. Such integrated approaches are essential to ensure both the conservation of houbara populations and the long-term sustainability of associated cultural practices such as falconry [73]. Figure 9 summarizes the five-stage conceptual flowchart of recommended mitigation strategies for Asian Houbara bustard (*Chlamydotis macqueenii*) conservation. As illustrated, Stage 1 (Genetic Profiling) must precede all subsequent interventions, as genetic compromise cannot be remedied through later-stage conditioning [81,105]. The adaptive feedback implicit in Stage 5 ensures that monitoring data inform iterative improvements to genetic, breeding, and behavioral protocols [73,76].

## 10. Future Research Direction and Recommendations

Numerous Asian Houbaras are bred in captivity through artificial insemination, with tens of thousands being released each year across most of the countries within the species’ habitat. Few land-dwelling species are managed in the wild with captive breeding on such a large scale, and the effects of this approach are not yet fully understood. Understanding how these releases either aid in establishing self-sustaining populations or, alternatively, diminish the sustainability of wild populations due to the introduction of captivity-adapted genotypes, competition for resources, or other factors, would be valuable for guiding conservation strategies [32].

The conservation of the Asian Houbara Bustard is one of the most intensive captive breeding efforts for a threatened bird species in the Middle East. Breeding programs set up by the National Wildlife Research Center (NWRC) in Saudi Arabia and the International Fund for Houbara Conservation (IFHC) in the UAE have shown that producing large numbers of captive houbara is technically possible. Saudi Arabia’s program achieved significant success, in terms of fertility rates rising from 69.5% to 90% using artificial insemination techniques between 1992 and 1998, and hatchability increasing from 53.7% to 75% during the same time [65]. These achievements highlight the potential of assisted reproductive technologies to improve production efficiency in captive settings.

However, the effectiveness of captive breeding as a solitary conservation strategy remains debated. As Burnside et al. [75] point out, there is considerable uncertainty regarding the ecological impacts of large-scale captive propagation programs. Four main concerns challenge the conservation value of these initiatives: artificial selection pressures in captive settings tend to favor phenotypic and genotypic changes, genetic integration of maladaptive traits into wild populations can occur through hybridization, supplementation can unintentionally increase hunting pressure and help predators learn, and high density conditions in captivity raise the risk of pathogen spread [76]. These issues are especially relevant for the houbara bustard, where introducing non-local genotypes might disrupt heritable migratory behavior [76,111].

The genetic risks of captive breeding have been well-documented in various species. Frankham [83] showed that genetic adaptation to captivity happens quickly, even when captive-bred individuals make up a small part of wild populations. This domestication process affects many traits, including morphology, physiology, endocrine function, and behavior, often with negative outcomes when reintroduced [83,84]. For the Houbara Bustard, released captive-bred individuals have shown deficiencies in foraging, avoiding predators, and territorial behavior, which often leads to lower survival and reproductive success compared to wild birds [94,97]. The loss of learned behaviors from parents during captive rearing further reduces reproductive success for these released birds [97,98].

The situation in Kuwait poses unique challenges and opportunities for Houbara conservation. Historical records show that Macqueen’s Houbara Bustard was once a breeding resident in Kuwait, with nesting activity documented in the early 20th century. Today, however, existing populations only visit in winter, with no confirmed breeding in recent decades [36]. The disappearance of breeding populations highlights the urgent need for conservation efforts and careful genetic sourcing of foundation stock. The Kuwait Institute for Scientific Research (KISR) should prioritize acquiring genetically suitable individuals for the Houbara Breeding Center (HBC) to avoid the risks of genetic homogenization.

Assisted reproductive technologies (ARTs) may provide promising ways to enhance houbara production and genetic management. Techniques like in vitro fertilization, surrogate shell culture, and primordial germ cell-mediated chimerism have been successfully used in related bird species [16,76,78] showed that using primordial germ cell technology with chicken embryos could produce pure-line houbara offspring, although production rates were still low. These methods might be particularly beneficial for Kuwait’s program by preserving genetic diversity and minimizing the number of captive generations, which should help reduce domestication effects [112].

FInduced pluripotent stem cells (iPSCs) hold significant potential in Houbara conservation by enabling non-invasive genetic preservation and advanced reproductive strategies. iPSC technology involves reprogramming somatic cells, such as feather pulp or blood, into a pluripotent state using integration-free methods like mRNA, Sendai virus, or episomal systems, which minimize genomic instability. This pluripotency allows differentiation into various cell types, including functional gametes through primordial germ cell-like intermediates, providing an alternative to traditional gamete collection and reducing reliance on donor oocytes. Additionally, blastoid formation from iPSCs offers a novel embryonic reconstruction pathway that can bypass seasonal reproductive limitations. Although challenges remain in lineage specification and maintaining epigenetic fidelity, integrating iPSC technology with ecosystem management can facilitate large-scale genetic rescue by converting cellular plasticity into population resilience, thus enhancing the genetic diversity and sustainability of Houbara populations [113].

Long-term genetic storage in cell biobanking is primarily facilitated by advanced cryopreservation technologies that stabilize biological samples at ultra-low temperatures, typically −196 °C in liquid nitrogen. This process halts all metabolic and biochemical activities, preserving cell viability indefinitely. The procedure begins with collecting viable cells or tissues, which are treated with cryoprotectants to prevent ice crystal formation that can damage cellular structures during freezing. Following cryoprotectant application, samples undergo controlled-rate cooling to minimize thermal and osmotic stress, further protecting cellular integrity. Once at cryogenic temperatures, samples are stored in specialized tanks that maintain consistent ultra-low temperatures, ensuring long-term stability. This approach allows reliable thawing and revival of genetic material for applications such as assisted reproductive technologies, breeding programs, and genetic research. By preserving genetic diversity, especially of endangered species like the Houbara, biobanking supports conservation efforts including population recovery, de-extinction, and gene editing. The integration of cryopreservation-based biobanking with emerging technologies like iPSC generation further expands the scope of genetic resource management by enabling the preservation and utilization of a broader range of cellular materials beyond traditional gametes and embryos [114].

The integration of captive breeding with habitat protection and sustainable use approaches is essential for long-term success. Dolman et al. [72] emphasize that sustainable hunting and conservation of threatened Houbara Bustards require integrated strategies that consider ecological and social factors. The Houbara’s status as an “*umbrella species*” has led to broader conservation benefits for desert ecosystems, yet the conflict between falconry practices and population recovery persists [41,51]. For Kuwait, the development of a comprehensive conservation strategy must engage stakeholders including falconry communities, policymakers, and regional conservation initiatives to ensure alignment of objectives and resource allocation.

Post-release monitoring remains a critical knowledge gap in Houbara conservation programs. While satellite telemetry has been employed to track migration patterns and survival rates [11,76], comprehensive demographic studies comparing wild and captive-bred individuals are limited. The IUCN/SSC [105] guidelines for reintroductions emphasize the necessity of robust monitoring to evaluate success and adapt management practices accordingly. Kuwait’s emerging program should prioritize the establishment of baseline ecological data and long-term monitoring protocols from inception to enable evidence-based adaptive management.

While captive breeding programs have achieved remarkable production success for the Asian Houbara Bustard, the translation of these efforts into self-sustaining wild populations remains uncertain. The scientific literature emphasizes that captive breeding should be embedded within comprehensive conservation strategies that address habitat protection, genetic management, behavioral conditioning, and post-release monitoring [73,81,105]. For Kuwait, the establishment of the Houbara Breeding Center represents an opportunity to implement these best practices from the outset, prioritizing genetic integrity, minimizing captive generations, and integrating with regional conservation networks to contribute to the long-term persistence of this culturally and ecologically significant species.

## 11. Conclusions

The Asian Houbara bustard (*Chlamydotis macqueenii*) is now listed as Vulnerable on the IUCN Red List. This status reflects ongoing population declines caused by habitat loss, hunting, and other human activities. Intensive captive breeding programs are essential for conserving the species. Their success relies on maintaining genetic diversity, pre-serving natural behavior, and integrating breeding efforts with larger ecological and conservation plans. Evidence shows that captive breeding by itself cannot guarantee population recovery. It can also create genetic and ecological risks if not managed well. In Kuwait, the creation of the Houbara Breeding Center offers a chance to follow best practices from the start. This includes choosing genetically suitable founder stock, limiting the number of captive generations, and conducting thorough post-release monitoring. When combined with habitat protection, engaging stakeholders, and flexible management strategies, captive breeding can significantly contribute to the long-term survival of the Asian Houbara bustard.

## Figures and Tables

**Table 1:** Morphological and ecological differences between African and Asian Houbara Bustards [30].

Features	Asian Houbara Bustard (*C. macqueenii*)	African Houbara Bustard (*C. undulata*)
Appearance	Larger, paler with a lighter brown back, and features black-tipped white crown feathers. Males have a pale blue-gray foreneck base and a white-based black neck plume.	Smaller, generally darker/duller coloration, with all-white crest feathers in the male. Males have a black-and-white peppered foreneck base and all-black neck plumes
Range	Distributed from the Sinai and Arabian Peninsula through to Mongolia and the Indian subcontinent.	Found in North Africa and the Canary Islands (subspecies *C. u. fuertaventurae*).
Crest	Contains longer, black feathers with white bases, which are more prominent during display.	The crest on the top of the head is primarily white.
Neck pattern	More prominent neck markings in males	Less distinct
Tail Pattern	Longer, paler	Shorter, darker
Legs	Pinkish-yellow	Yellowish
Habitat	Steppe and semi-desert from Middle East to Central Asia	Steppe, semi-desert in North Africa
Behavior	Highly migratory, moving from Central Asia to warmer regions for winter.	Mostly sedentary, with limited migration.
Subspecies	sometimes referred to as the Asian race of *C. undulata* historically	*C. u. undulata*, *C. u. fuerteventurae*

**Table 2:** Data on Houbara Bustard’s conservation during the last 30 years (1995–2024).

Serial Number	Global Status of the Houbara Bustard	Conservation Organizations	Conservation Status and Breeding
1	Endangered ^1^	National Wildlife Research Center (NWRC) in Taif, Saudi Arabia.	The release of captive-bred Houbara into protected areas
2	Vulnerable ^2^	National Wildlife Research Center (NWRC) in Taif, Saudi Arabia.	Compression between wildcaught and captive-reared birds.
3	Endangered ^3^	International Fund for Houbara Conservation (IFHC), UAE	The reintroduced population of the captive-bred
4	Vulnerable ^4^	International Fund for Houbara Conservation (IFHC), UAE	Strike a balance between Houbara preservation and falconry
5	Vulnerable ^5^	Al Baida Research Centre for Houbara and Bird Breeding, Qatar	Intensive conservation through captive breeding

Sources: ^1^ [68], ^2^ [69], ^3^ [70], ^4^ [71], ^5^ [64].

## Data Availability

No new data were created or analyzed in this study. Data sharing is not applicable to this article.

## Abbreviations

ART	Assisted Reproductive Technologies
CITES	Convention on International Trade in Endangered Species of Wild Fauna and Flora
ESU	Evolutionarily Significant Unit
HBC	Houbara Breeding Center
IFHC	International Fund for Houbara Conservation
iPSCs	Induced pluripotent stem cells
IUCN	International Union for Conservation of Nature
KISR	Kuwait Institute for Scientific Research
NCWCD	National Commission for Wildlife Conservation and Development
NWRC	National Wildlife Research Center
UAE	United Arab Emirates
